# *Pleurotus eryngii* Stipe Base-Derived Carbon Dots Enhanced the Growth and Salt Tolerance of Tomato

**DOI:** 10.3390/plants14203227

**Published:** 2025-10-20

**Authors:** Xu Zhang, Yi Gao, Wenhui Wang, Hao Wang, Yu Xin, Rongrui Kang, Wenfeng Nie, Han Du, Qinghua Shi

**Affiliations:** 1 State Key Laboratory of Crop Biology, College of Horticulture Science and Engineering, Shandong Agricultural University, Taian 271018, China; zhangxu@sdau.edu.cn (X.Z.);; 2 College of Food Science and Engineering, Shandong Agricultural University, Taian 271018, China

**Keywords:** tomato, growth, salt stress, carbon dots, antioxidants

## Abstract

Soil salinity severely limits tomato growth by impairing photosynthesis and redox balance. Carbon dots (CDs) have emerged as promising nanomaterials to promote growth and enhance stress tolerance. In this study, we synthesized and characterized CDs derived from *Pleurotus eryngii* stipe bases (PbCDs), with rich hydrophilic groups including carboxyl groups and secondary amines. The particles were uniform, with an average diameter of 4.17 nm, and exhibited blue fluorescence. Importantly, PbCDs significantly promoted tomato growth under control and salt conditions. Under salt conditions, PbCD-treated plants showed enhanced shoot and root growth, larger leaf area, and growth comparable to control plants without stress. Interestingly, PbCD treatment of the plants enhanced cell expansion under control conditions and promoted cell division under salt conditions. In addition, PbCD-treated plants had higher chlorophyll content, net photosynthetic rate, and maximum quantum efficiency of PSII, which alleviated the inhibition caused by salinity. Furthermore, PbCDs also reduced oxidative damage by lowering O_2_^•−^, and H_2_O_2_ levels, while activating antioxidant enzymes (superoxide dismutase, catalase, peroxidase, and ascorbate peroxidase) under salt stress. Overall, PbCDs promoted tomato growth and conferred salt tolerance through coordinated regulation of the cell cycle, photosynthesis, and antioxidant defenses, supporting their potential as sustainable nanomaterials for crop improvement in saline soils.

## 1. Introduction

Crop yield is significantly restricted by soil salinity, which affects nearly 6% of global arable land [1]. Salinity imposes hyperosmotic stress on plant cells due to reduced water availability, thereby disrupting the cellular redox balance and resulting in excessive production of reactive oxygen species (ROS) [2]. Excessive ROS accumulation can lead to oxidative damage of cellular structures and promote lipid peroxidation. However, plants have evolved a sophisticated enzymatic antioxidant defense system, including superoxide dismutase (SOD), catalase (CAT), peroxidase (POD), and ascorbate peroxidase (APX), to scavenge ROS and maintain redox homeostasis under salinity stress [2].

Nanomaterials have emerged as promising tools in modern agriculture, offering new strategies to enhance crop productivity and stress tolerance [3]. Various nanoparticles, including metal oxides and carbon-based nanomaterials, have been applied to promote plant growth, improve fertilizer use efficiency, and strengthen resistance against biotic and abiotic stresses [4,5,6]. Among them, carbon dots (CDs), a new class of carbon nanomaterials with sizes below 10 nm, have attracted increasing attention due to their unique advantages, such as large specific surface area, excellent photoluminescence, biocompatibility, and low toxicity [7]. Compared with conventional metal-based nanoparticles, CDs are considered safer and more environmentally friendly, making them particularly suitable for agricultural applications [8]. Recent studies have demonstrated that CDs can accelerate seed germination, promote root and shoot elongation, enhance photosynthesis, and improve nutrient uptake in crops [4,7,9,10]. Moreover, CDs also act as ROS scavengers, thereby alleviating oxidative damage and enhancing plant tolerance to drought and salinity [11,12].

Biomass-derived CDs are especially attractive, because they can be synthesized from abundant agricultural and food processing residues, which reduces waste and adds value to underutilized resources [13]. In addition, mushroom stipe bases, which are often discarded during edible mushroom cultivation, represent a typical waste biomass rich in organic carbon and functional groups, making them an excellent raw material for green CD synthesis [14]. Therefore, utilizing mushroom stipe base not only addresses the issue of agricultural waste management but also provides a sustainable and low-cost route for producing functional nanomaterials. The *Pleurotus eryngii* (*P. eryngii*), a popular mushroom, is characterized by thick flesh, a tender and crisp texture, and an attractive appearance. However, whether CDs derived from *P. eryngii* stipe bases can be synthesized and applied to regulate plant growth and stress tolerance remains unclear.

Tomato (*Solanum lycopersicum* L.) are a major fruit vegetable crop with considerable nutritional and economic importance, and also provide a representative system for evaluating agricultural applications. Nevertheless, abiotic stresses including salinity drastically suppress plant growth and development, thereby limiting production [2]. In this study, we synthesized and comprehensively characterized a *P. eryngii* stipe base-derived CD (PbCDs) and evaluated its role in promoting tomato growth and enhancing salt-stress tolerance. Collectively, this study not only broadens the understanding of plant–nanomaterial interactions but also establishes a theoretical and practical basis for developing environmentally friendly strategies to enhance crop productivity under abiotic salt stress.

## 2. Results

### 2.1. Structural and Morphologic Characterization of PbCDs

**Figure 1:**
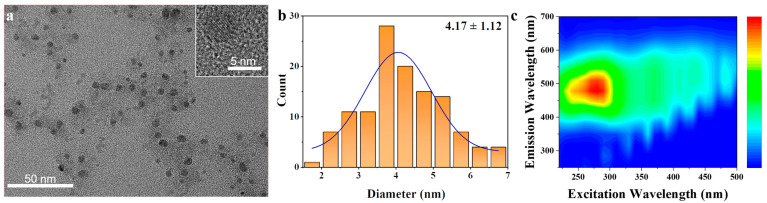
Verification of the morphological characteristics, and fluorescence properties of *Pleurotus eryngii* (*P. eryngii*) stipe base-derived CDs (PbCDs). (**a**) Transmission electron microscope (TEM) result. The scale bar represented 50 nm. (Inset: High resolution TEM image with a scale bar representing 5 nm). (**b**) Size distribution of PbCDs. (**c**) Fluorescence 3D scanning spectrum of PbCDs.

We selected the stipe base of *P. eryngii* to synthesize CDs through heat treatment. The morphology of the obtained PbCDs was characterized by transmission electron microscopy (TEM). As shown in Figure 1a, the PbCDs exhibited high monodispersity. The particle size distribution ranged from 1.91 to 6.73 nm, with an average diameter of (4.17 ± 1.12) nm (Figure 1b). The three-dimensional fluorescence spectrum (Figure 1c) was scanned to reveal that the aqueous dispersion of PbCDs emitted strong blue fluorescence under excitation wavelengths ranging from 220 nm to 470 nm, indicating excitation-independent photoluminescence behavior. The optimal excitation and emission wavelengths were observed at 285 nm and 485 nm, respectively.

**Figure 2:**
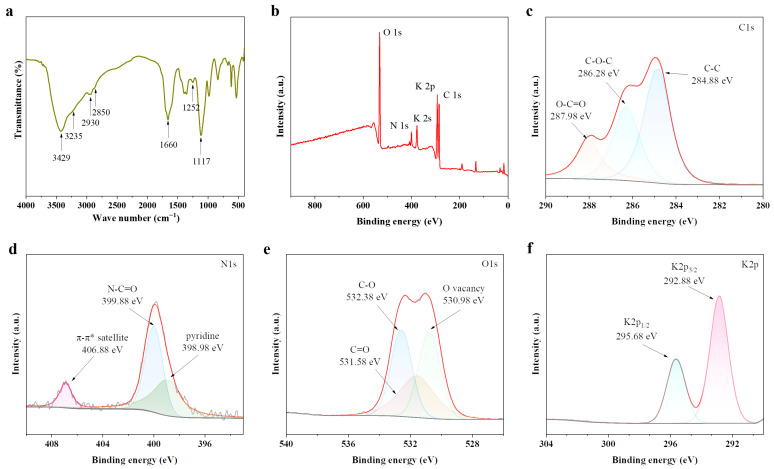
Characterization of elemental groups of PbCDs. (**a**) Fourier transform infrared spectroscopy (FT-IR) spectra. (**b**) X-ray photoelectron spectroscopy (XPS) spectra in high resolution of C1s (**c**), N1s (**d**), O1s (**e**), and K2p (**f**) of PbCDs.

The surface functional groups of the PbCDs were analyzed by Fourier transform infrared (FT-IR) spectroscopy. As shown in Figure 2a, a strong absorption band around 3429 cm^−1^ corresponded to O–H stretching vibrations, while the peak at 1117 cm^−1^ indicated C–O bending vibrations. The band at 3235 cm^−1^ was attributed to N–H stretching. Characteristic symmetric and asymmetric stretching vibrations of –CH_2_– groups were identified at 2850 cm^−1^ and 2930 cm^−1^, respectively. A sharp peak at 1660 cm^−1^ may be assigned to C=O or C=N stretching vibrations. Additionally, the absorption at 1252 cm^−1^ was associated with N–H bending or C–N stretching modes. Peaks in the fingerprint region suggested the existence of aromatic rings.

X–ray photoelectron spectroscopy (XPS) survey spectra confirmed that PbCDs were primarily composed of carbon, nitrogen, oxygen, and potassium (Figure 2b). High-resolution spectra provided further insight into the bonding environments of these elements. The C1 spectrum (Figure 2c) was fitted with three component peaks at 284.88 eV, 286.28 eV, and 287.98 eV, assigned to C–C/C=C, C–O, and O–C=O bonds, respectively. The N1s spectrum (Figure 2d) revealed two major peaks at 398.98 eV and 399.88 eV, with characteristics of pyridinic nitrogen and amide (N–C=O) functionalities. Additionally, a weak satellite feature at 406.88 eV, associated with π–π* transitions in nitrogen-containing aromatic systems, was also observed, consistent with FT-IR findings. The O1 spectrum (Figure 2e) exhibited contributions from oxygen vacancies (530.98 eV), C–O (531.58 eV), and C=O (532.38 eV). Since potassium has no obvious absorption in the FT-IR range, its existence cannot be confirmed by infrared spectroscopy. In XPS, the weak K2p peak is easily confused with the C1 (CF_2_) peak or C1 satellite structure of aromatic compounds. The K2 peak was found at 378 eV to support the presence of potassium, and the K2p peak in Figure 2f exhibited a spin–orbit splitting feature of 2.8 eV.

### 2.2. PbCD Treatment Promoted Tomato Growth and Enhanced Tomato Salt Tolerance

**Figure 3:**
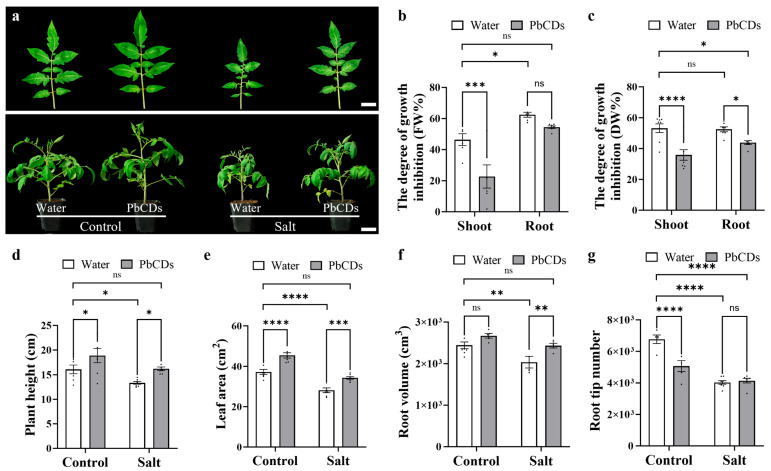
PbCD treatment promoted tomato growth and enhanced tomato salt tolerance. (**a**) Phenotype of control and PbCD-treated tomato plants under control and salt treatments (scale bar: 3 cm). The degree of growth inhibition (FW%) (**b**), the degree of growth inhibition (DW%) (**c**), plant height (**d**), leaf area (**e**), root volume (**f**), and root tip number (**g**) were measured in the indicated plants. Data was the mean ± SD from at least four biological replicates. Two-way ANOVA followed by Tukey’s test was used for significance analysis. *, **, ***, and **** indicated significant differences at *p* < 0.05, *p* < 0.01, *p* < 0.001, and *p* < 0.0001, respectively, and “ns” represented “no significant difference”.

We investigated the function of PbCDs on the regulation of tomato growth and salt tolerance. As shown in Figure 3a, PbCD-treated tomato plants displayed better growth states than water-treated tomato plant in both control and salt conditions. Notably, PbCD-treated tomato plants in salt conditions showed growth similar to the water-treated tomato plants in control conditions. Importantly, under long-term salt stress, the shoot and root growth of all tomato plants was severely suppressed (Figure 3a,b). However, the degree of growth inhibition due to salt exposure was significantly reduced in the PbCD-treated tomato plants (Figure 3a,b). In addition, except for root tip number, plant height, leaf area, and root volume were significantly increased in PbCD-treated tomato plants in both control and salt conditions (Figure 3d–g). Interestingly, the growth indexes of the tomato plants treated with PbCDs in salt conditions also recovered similarly to the water-treated tomato plants in control conditions. These data indicated that PbCD treatment enhanced the growth and salt tolerance of tomato.

### 2.3. PbCD Treatment Affected Tomato Cell Expansion and Division by Altering Cell Cycle Progression Under Control and Salt Conditions

**Figure 4:**
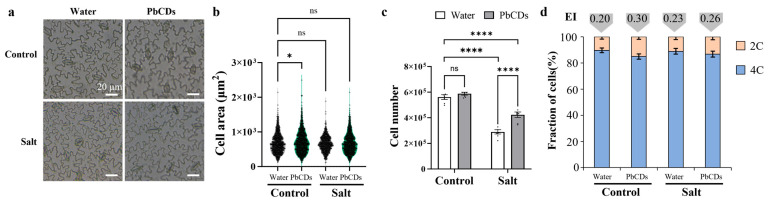
PbCD treatment affected tomato cell expansion and division by altering cell cycle progression under control and salt conditions. (**a**) Cell phenotype in the indicated plant leaves (Scale bar represented 20 μm). (**b**–**d**) represented cell area, cell number, and fraction of cells in the indicated plant leaves, respectively. Endocycle index (EI) in (**d**) represented endocycle extent. Data was the mean ± SD from at least four biological replicates. One-way ANOVA followed by Tukey’s test was used for significance analysis of cell area. Two-way ANOVA followed by Tukey’s test was used for significance analysis of cell number. * and **** indicated significant differences at *p* < 0.05 and *p* < 0.0001, respectively, and “ns” represented “no significant difference”.

Given that PbCD treatment promoted tomato growth in both control and salt conditions (Figure 3), the cell areas and cell numbers of the tomato leaves were measured. As shown in Figure 4a–c, under control conditions, the cell area was significantly increased in PbCD-treated tomato leaves and the cell number was not significantly different between water-treated tomato leaves and PbCD-treated tomato leaves. Interestingly, under salt conditions, the cell area showed no significant difference between the water-treated tomato leaves and PbCD-treated tomato leaves, but the cell number was significantly increased in PbCD-treated tomato leaves. Because cell expansion and division were regulated by cell cycle, we measured the fraction of cells in all tomato leaves and analyzed their endocycle extent (Figure 4d). As expected, under control conditions, compared with those in water-treated tomato leaves, the 2C fraction of cells in PbCD-treated tomato leaves was obviously decreased, and the 4C fraction of cells in PbCD-treated tomato leaves was decreased. In addition, the endocycle index was significantly increased in PbCD-treated tomato leaves. Under salt conditions, the 2C and 4C fractions of cells and endocycle indices showed similar percentages in water- and PbCD-treated tomato leaves.

### 2.4. PbCDs Enhanced Photosynthetic Capacity, Thereby Promoting Tomato Growth and Increasing Salt Tolerance

**Figure 5:**
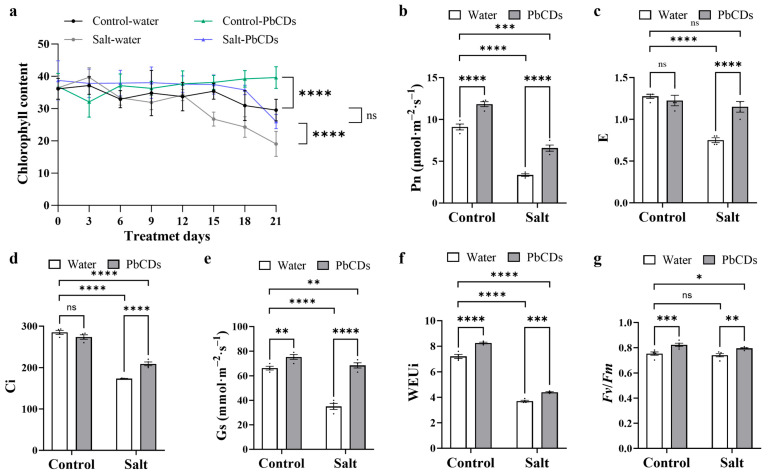
PbCD treatment strengthened the photosynthetic ability of tomato under saline stress. (**a**–**g**) represented chlorophyll content, net photosynthetic rate (Pn), transpiration rate (E), intercellular CO_2_ concentration (Ci), stomatal conductance (Gs), intrinsic water-use efficiency (WEUi), and the maximum quantum yield of photosystem II (*Fv*/*Fm*) in the indicated plant leaves, respectively. Data was the mean ± SD from at least four biological replicates. Two-way ANOVA followed by Tukey’s test was used for significance analysis. *, **, ***, and **** indicated significant differences at *p* < 0.05, *p* < 0.01, *p* < 0.001, and *p* < 0.0001, respectively, and “ns” represented “no significant difference”.

Next, we assessed the photosynthetic capacity in control-treated and PbCD-treated tomato plants. As shown in Figure 5a, as time progressed, the chlorophyll content was markedly decreased under salt conditions but was significantly enhanced in all PbCD-treated plants’ leaves compared with those of water-treated plants. In addition, except for transpiration rate (E) and intercellular CO_2_ concentration (Ci), net photosynthetic rate (Pn), stomatal conductance (Gs), intrinsic water-use efficiency (WEUi), and the maximum quantum efficiency of PSII (*Fv*/*Fm*) were significantly increased in PbCD-treated tomato plants compared with those of water-treated tomato plants under control conditions (Figure 5b–g). However, under salt conditions, all of these parameters displayed significant enhancement in PbCD-treated tomato plants compared with water-treated tomato plants. These results meant that the salt stress induced depression in photosynthesis of tomatoes was alleviated by PbCD treatment.

### 2.5. PbCD Treatment Alleviated Salt Stress-Induced Oxidative Stress and Activated the Antioxidant Enzyme Activity in Tomato

**Figure 6:**
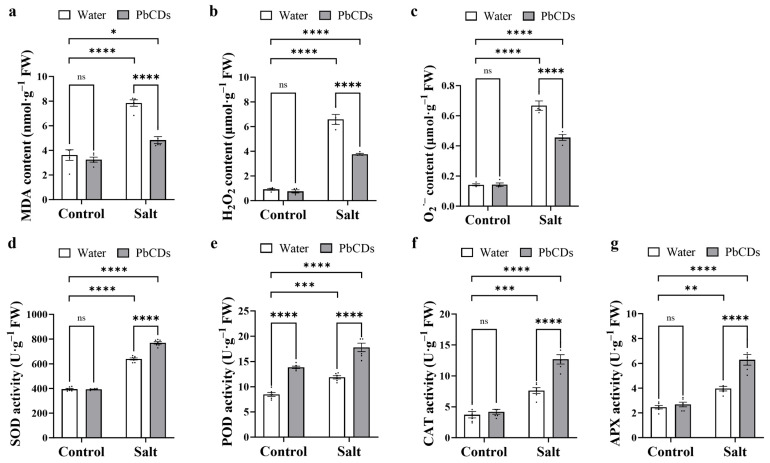
PbCD treatment reduced the content of malondialdehyde (MDA), H_2_O_2_, and O_2_^•−^, and increased the antioxidant enzymatic activities in tomato. (**a**–**g**) represented MDA content, H_2_O_2_ content, O_2_^•−^ content, superoxide dismutase (SOD) activity, peroxidase (POD) activity, catalase (CAT) activity, and ascorbate peroxidase (APX) activity in the indicated plant leaves, respectively. Data was the mean ± SD from at least four biological replicates. Two-way ANOVA followed by Tukey’s test was used for significance analysis. *, **, ***, and **** indicated significant differences at *p* < 0.05, *p* < 0.01, *p* < 0.001, and *p* < 0.0001, respectively, and “ns” represented “no significant difference”.

For analyzing the aggregation of lipid peroxidation induced by salt stress, malondialdehyde (MDA) content was measured. As shown in Figure 6a, under control conditions, the content of MDA showed no significant difference between PbCD-treated tomato plants and water-treated tomato plants. However, under salt conditions, the content of MDA significantly decreased in PbCD-treated tomato plants. Furthermore, the levels of O_2_^•−^ and H_2_O_2_ were measured. As shown in Figure 6b,c, under control conditions, the contents of O_2_^•−^ and H_2_O_2_ showed no significant difference between water-treated and PbCD-treated tomato plants’ leaves. Nevertheless, under salt conditions, the contents of O_2_^•−^ and H_2_O_2_ were significantly reduced in PbCD-treated tomato plants’ leaves.

To evaluate the PbCD-mediated ROS scavenging ability of tomato exposed to salt treatment, we determined four antioxidant enzyme activities. As shown in Figure 6d–g, under normal conditions, compared with water-treated tomato leaves, only POD activity was significantly increased in CD-treated leaves, whereas the activities of SOD, CAT, and APX showed no significant differences between the two treatments. Notably, under salt conditions, the activities of SOD, POD, CAT, and APX were significantly more activated in PbCD-treated tomato plants compared with water-treated tomato plants.

### 2.6. Correlation Analysis of Physiological Parameters of Tomato Treated with PbCDs Under Salt Stress

**Figure 7:**
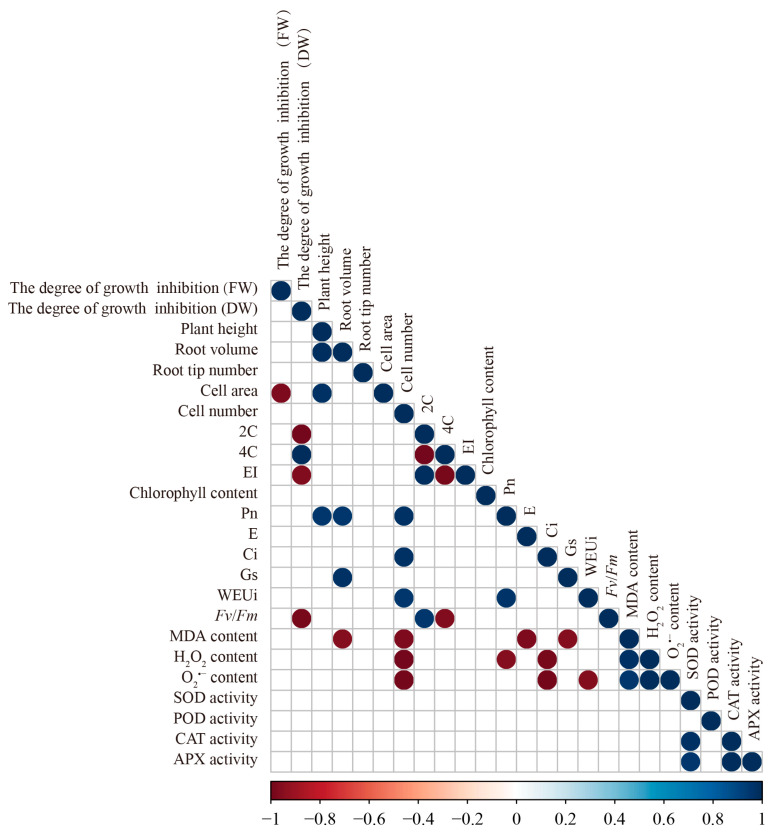
Correlation analysis of growth and physiological indices in PbCD-treated tomato plants under salt stress.

To identify the relationship between growth and physiological parameters in tomato plants treated with PbCDs under salt stress, a correlation analysis was performed (Figure 7). The degree of growth inhibition based on FW was significantly and negatively correlated with cell area. Besides, the DW inhibition showed a significant positive correlation with 4C content, while were negatively correlated with 2C proportion, EI, and *Fv*/*Fm*. In addition, plant height was positively correlated with root volume, cell area, and Pn. Root volume was positively correlated with Pn and Gs, and negatively correlated with MDA content. Cell numbers exhibited a positive correlation with Pn, Ci, and WEUi, but exhibited a negative correlation with the content of MDA, H_2_O_2_, and O_2_^•−^. Moreover, positive correlations were observed among MDA, H_2_O_2_, and O_2_^•−^ contents. In addition, strong positive correlations were also detected among antioxidant enzyme activities (SOD, CAT, and APX). These results revealed the distinct correlation patterns among physiological traits, reflecting the interdependence between growth performance, photosynthetic characteristics, oxidative status, and cellular properties in PbCD-treated tomato plants under salt stress.

## 3. Discussion

Biomass-derived CDs have been prepared from various edible mushrooms using various methods, which typically involve a carbonization step, and have been widely applied in imaging, detection, and food preservation [15,16,17,18]. The synthesized CDs often exhibit blue fluorescence [19], which is consistent with the results of this study. The uniform particle size and excitation-independent fluorescence characteristic of PbCDs (Figure 1) enabled treated tomatoes to absorb more blue light under sunlight exposure, thereby promoting plant growth in some ways [20]. Most studies have found that CDs derived from edible mushrooms are primarily composed of C, N, and O elements [21,22]. Similarly, these three major elements have also been identified in the PbCDs by FT-IR and XPS analysis (Figure 2). The absorption peaks of C–O, C–H, N–H, and C=O in Figure 2a, as well as the consistent results of XPS spectra (Figure 2c–e), proved that the surface of PbCDs contains a large number of secondary amines and carboxyl groups, endowing them with high hydrophilicity. Additionally, the presence of oxygen vacancies (Figure 2e) might provide more sites and activity for potential redox or catalytic interactions between PbCDs and plants [23]. Interestingly, the PbCDs also contained a considerable amount of potassium (Figure 2f), which can be attributed to the inherently high potassium content in *P. eryngii* [24]. Potassium is an essential macronutrient for the growth, development and stress tolerance of plant [25]. These might explain the effects of growth and salt tolerance of PbCDs on tomato in this study.

Salt stress is a major abiotic factor that severely restricts tomato growth by suppressing shoot and root development, thereby reducing overall plant productivity [26]. Our results demonstrated that PbCDs significantly promoted tomato growth under both control and salt conditions, as indicated by increased plant height, leaf area, and root volume (Figure 3). Notably, the growth status of PbCD-treated plants under salinity was comparable to that of control plants without stress, suggesting that PbCDs effectively alleviated the growth inhibition induced by salt stress. These results indicated that PbCDs not only enhanced plants’ growth but also conferred a protective effect that maintains growth performance under long-term stress. Plant growth ultimately depends on both cell expansion and cell division, processes that are tightly regulated by cell cycle [27,28]. At the cellular level, PbCDs influenced both cell expansion and division in tomato leaves through modulation of cell cycle progression (Figure 4). Under control conditions, PbCDs enlarged cell area and promoted endoreduplication, as reflected by the decreased 2C fraction and elevated endocycle index. These changes implied that PbCDs facilitated endocycle entry, thereby enhancing cell expansion and organ growth. In contrast, under salt stress, PbCDs did not significantly alter cell area but increased cell numbers, suggesting a shift toward promoting mitotic activity to compensate for salt-stress-induced growth restriction. This differential regulation meant that the plasticity of PbCDs in modulating growth pathways depends on environmental changes. Overall, these results suggested that PbCDs alleviated the inhibition of tomato growth caused by salt stress not only by improving plant morphological traits but also by modulating the balance between cell expansion and division through precise regulation of the cell cycle. The ability of PbCDs to shift between promoting endoreduplication under favorable conditions and stimulating cell division under salt conditions indicated their role as versatile regulators of plant growth and stress adaptation.

Salt stress is known to impair photosynthetic performance by inducing osmotic imbalance and reducing Ci, thereby limiting chlorophyll accumulation, PSII efficiency, and Pn [29]. Indeed, Ci, Gs, chlorophyll content, and Pn were significantly decreased under salinity, indicating damage to the photosynthetic apparatus (Figure 5). Notably, PbCD treatment alleviated this inhibition, and the photosynthetic capacity of PbCD-treated plants under salt stress was restored nearly to the control level. Similar promoting effects of CDs on photosynthesis have been reported previously. For example, CDs with optimized quantum yield were shown to convert harmful UV into photosynthetically active radiation, thereby improving PSII efficiency, electron transport rate, chlorophyll content, and RuBisCO activity in rice [30]. Likewise, CDs derived from natural biomass enhanced yield and nutritional quality in lettuce and tomatoes by stimulating mineral uptake and photosynthesis. These studies are consistent with our results, indicating that PbCDs promote CO_2_ assimilation and energy conversion efficiency to sustain tomato growth under saline conditions.

Another major consequence of salt stress is the excessive accumulation of ROS, including O_2_^•−^ and H_2_O_2_, which causes oxidative damage by disrupting membranes and deactivating enzymes [2]. We found that PbCD treatment significantly reduced O_2_^•−^ and H_2_O_2_ levels, as well as the lipid peroxidation marker MDA (Figure 6a–c), thereby protecting cellular membranes from oxidative injury. Comparable effects have been documented for Salvia miltiorrhiza-derived CDs, which not only scavenged ROS but also mobilized Ca^2+^ signaling to reinforce plant adaptation under salinity [6]. These findings suggested that ROS scavenging is a common mechanism by which biomass-derived CDs alleviate abiotic stress. In addition, the regulation of ROS homeostasis relies on antioxidant defenses. Enhanced SOD, CAT, POD, and APX activities are essential for ROS detoxification under salinity [1,31], and we observed that PbCDs strongly activated these enzymes in salt-stressed tomatoes (Figure 6d–g). However, under control conditions, only POD activity was significantly increased, suggesting that PbCDs preferentially induce antioxidant responses when ROS accumulation is triggered. Similar induction of antioxidant activity was also reported for functional carbon nanodots, which improved tomato tolerance in saline–alkali soils by stimulating antioxidant enzymes, osmotic adjustment, and nutrient uptake, in addition to enhancing photosynthesis [11,12]. These studies confirmed that the activation of antioxidants is a general and beneficial trait of CDs across plant systems.

Together, our results demonstrated that *P. eryngii* stipe base-derived PbCDs enhance tomato salt tolerance through a dual mechanism: by promoting photosynthetic efficiency and reinforcing antioxidant defenses to mitigate oxidative damage. By integrating ROS scavenging, enzymatic activation, and photosynthetic enhancement, PbCDs act as both protectors against stress-induced oxidative damage and stimulators of energy metabolism. These findings expanded on earlier research with biomass-derived CDs and emphasized the potential of PbCDs as sustainable nanomaterials for improving crop tolerance and productivity in saline soils.

## 4. Materials and Methods

### 4.1. The Synthesis and Characterization of PbCDs

The preparation of PbCDs was achieved by heat treatment as follows: 1 g *P. eryngii* stipe base was placed in a crucible and treated at 250 °C for 6 h in a muffle furnace. The resulting solid was then ground fine with a mortar and pestle and 20 mL of ultrapure water was added to it. The mixture was ultrasonicated for 30 min and then centrifuged at 10,000 rpm for 10 min, followed by dialysis for 24 h (1 kDa), and the solution was stored in the dark at 4 °C. The morphological and size characteristics were examined by TEM (JEM-F200 JEOL, Tokyo, Japan); fluorescence properties were assessed with a fluorescence spectrometer (LF-1701009, Thermo Fisher Scientific, Waltham, MA, USA); and surface functional groups and elemental composition were analyzed by FT-IR (NICOLET iS20, Thermo Fisher Scientific, Waltham, MA, USA) and XPS (K-Alpha, Thermo Fisher Scientific, Waltham, MA, USA). *P. eryngii* was provided by Professor Yan Zhang from the College of Plant Protection, Shandong Agricultural University.

### 4.2. Plant Materials Condition

The tomato seeds (*Solanum lycopersicum* L. cv. Heinz 1706) germinated and grew in the growth chamber at Shandong Agricultural University with a 16 h light (28 °C)/8 h dark (22 °C) photoperiod, as previously described [32]. In detail, to disinfect the surface of the seeds, they were soaked in 75% ethanol for 1 min and then treated with 8% NaClO solution for 15 min. Then, the seeds were rinsed five times with sterile water and sown in a medium containing Morishige and Skoog (MS) with added sucrose. In a pot of 8 × 8 × 8.5 cm, dry seeds were directly sown at different depths. The soil contained 40% organic matter.

### 4.3. Salt Treatment

For salt treatment, three-week-old tomato plants were treated with 100 mM NaCl. Salt treatments were applied at three-day intervals with three applications. Then, plants were sprayed with 50 mg·mL^−1^ PbCD solution or water at four-day intervals with five applications. Four days later, related indicators of these plants were collected. For the control treatment, water and PbCDs were applied in the same way as under the salt treatment. Plants were photographed, and then the plant height and the 5th leaf area were determined at the indicated time points using Image J v154 software (https://imagej.net/ij/, accessed on 16 October 2025). Tomato leaves were flash-frozen, ground to powder, and stored at −80 °C. All measurements were performed with at least four biological replicates.

### 4.4. Degree of Growth Inhibition Analysis

The degree of growth inhibition was analyzed according to the method described in our previous study [1]. The degree of growth inhibition (FW/DW) was calculated using the following formula: degree of growth inhibition (%) = (Control − Salt)/Control × 100%. The fresh weight (FW) of the shoots was recorded immediately. For dry weight (DW), samples were first heated at 105 °C for 20 min, followed by drying at 75 °C until a constant weight was reached. All measurements were conducted in three independent experiments, with at least four biological replicates.

### 4.5. Cell Area, Cell Number, and DNA Ploidy Analysis

To determine cell area and cell number, the abaxial epidermises of the 5th leaf of six-week-old tomato plants were collected. Then, images of cell phenotypes were visualized using a microscope and measured by Image J v154 software.

For DNA ploidy measurement, the 5th leaves of these indicated tomato lines were used for DNA ploidy analysis. Leaf samples were chopped in an ‘Aru’ buffer, filtered, and stained with propidium iodide based on the previous description [33]. More than 8000 nuclei in each sample were measured by a BD flow cytometer. At least three biological replicates were analyzed in these tomato plants. To quantize endocycle extent, Endocycle indices (EI) were calculated with the following formula: EI = (%4C nuclei × 1) + (%8C nuclei × 2) + (%16C nuclei × 3) + (%32C nuclei × 4). All measurements were conducted in three independent experiments, with at least four biological replicates.

### 4.6. Photosynthetic-Related Parameter Analysis

Chlorophyll was extracted from 0.05 g tomato leaf tissue by 80% acetone and was measured using a chlorophyll meter (SPAD-502 Plus, Konica Minolta, Tokyo, Japan) at OD_470_, OD_647_, and OD_663_. The chlorophyll content was calculated according to Formulae (1–3):

(1) $$chla\left(mg/L\right)=12.21\times {OD}_{663}-2.81\times {OD}_{647}$$

(2) $$chlb\left(mg/L\right)=20.13\times {OD}_{647}-5.03\times {OD}_{663}$$

(3) $$Total\;chl\left(mg/L\right)=\frac{1000\times {OD}_{647}-3.27\times chla-104\times chlb}{229}$$

The Pn, Ci, E, Gs, and WEUi of the 5th leaf was determined with a CIRAS-3 Portable Photosynthesis System using the following settings: ambient CO_2_ (360 ppm), photon flux density (PFD) of 500 μmol·m^−2^·s^−1^, vapor pressure vapor (VPD) of 1.9–2 kPa, and air temperature 25 ± 1 °C. In addition, the *Fv*/*Fm* was measured by a Pulse Modulated Fluorometer (FMS-2, Hansha Scientific Instruments Limited, Taian, China). All measurements were conducted in three independent experiments, with at least four biological replicates.

### 4.7. Assessment of Resistance-Related Indicators

Lipid peroxidation was evaluated by measuring the levels of O_2_^•−^ and H_2_O_2_ and MDA content in tomato leaves as described by the article we published previously [34].

The content of O_2_^•−^ was extracted from 0.05 g of tomato leaf tissue using a commercial assay kit (BC1295, Beijing Solarbio Science & Technology Co., Ltd., Beijing, China) and quantified by measuring absorbance at 530 nm. The concentration was calculated using Equation (4), where C_standard_ = 0.03125 μmol·mL^−1^, V = 0.1 mL, and W = 0.05 g.

The H_2_O_2_ content was also determined by extracting 0.5 g of tomato leaf tissue using another commercial assay kit (BC3595, Beijing Solarbio Science & Technology Co., Ltd.), with absorbance measured at 415 nm. The calculation was also performed using Equation (4), with C_standard_ = 2 μmol·mL^−1^, V = 0.1 mL, and W = 0.05 g.

(4) $$Content=2\times C_{standard}\frac{A_{sample}-A_{blank}}{A_{standard}-A_{blank}}\times \frac{V}{W}$$

The crude enzyme extract for MDA, SOD, CAT, and APX analyses was prepared by homogenizing 0.1 g of leaf tissue in 0.05 mol·L^−1^ phosphate buffer (pH 7.8), followed by centrifugation to collect the supernatant.

To assay MDA content, 300 μL of crude enzyme extract was mixed with 0.67% thiobarbituric acid (TBA), and the absorbance at 450, 532, and 600 nm was measured. The calculation formula for MDA content was as follows:

(5) $$MDA\;content=C\times \frac{V}{W}\;$$

where C = 6.45 × (A_532_ − A_600_) − 0.56 × A_450_, V = 3 mL, and W = 0.1 g.

In addition, the enzymatic activities of SOD, CAT, and APX in tomato leaves were assessed according to established protocols [1]. In detail, SOD activity was assayed by adding 25 μL of the crude enzyme extract to 18 μL of 0.6 mg·mL^−1^ nitroblue tetrazolium (NBT) solution and monitoring absorbance at 560 nm. The activity was calculated according to Equation (6), with Alight = absorbance of the light control, Adark = absorbance of the dark control, A = absorbance of the sample, W = 0.1 g, V = 0.6 mL, and V_t_ = 0.025 mL:

(6) $$SOD\;content=\left(A_{light}-A+A_{dark}\right)\times \frac{V}{{0.5\times A}_{light}\times W\times V_{t}}\;,$$

CAT activity was determined by incubating 5 μL of crude enzyme extract with 50 μL of hydrogen peroxide at a concentration of 0.1 mol/L and then monitoring the decrease in absorbance at 240 nm, as calculated by Equation (7). APX activity was determined by adding 5 μL of crude enzyme extract to 100 μL of 0.88 mg/mL ascorbic acid hydrogen peroxide and then measuring the absorbance at 560 nm, which was calculated as follows:

(7) $$Activity=\frac{∆A\times V}{a\times W\times t}\;$$

where ΔA = [|A_t1_ − A_t0_| + |A_t2_ − A_t1_| + … + |A_t15_ − A_t14_|]/15, V = 0.6 mL, a = 0.01 mL, W = 0.1 g, and t = 1 min.

All measurements were conducted in three independent experiments, with at least four biological replicates.

### 4.8. Data Analysis

Correlation analysis was performed and visualized in R v4.0.3 software using the corrplot package. All data were compiled in Excel, and statistical analyses were conducted using GraphPad Prism 10. Means and SD were calculated from multiple replicates. One-way or two-way ANOVA followed by Tukey’s post hoc test were applied to determine statistical significance. Results were expressed as mean ± SD from at least four biological replicates. Significance levels were indicated as *, **, ***, and **** corresponding to *p* < 0.05, *p* < 0.01, *p* < 0.001, and *p* < 0.0001, respectively.

## 5. Conclusions

In this study, we synthesized and characterized CDs derived from *P. eryngii* stipe base and demonstrated their growth-promoting and stress-alleviating effects in tomato plants. PbCDs exhibited uniform size, strong blue fluorescence, and abundant functional groups, with unique potassium enrichment potentially contributing to their biological activity. Importantly, PbCDs significantly enhanced shoot and root growth, leaf expansion, and root development under both control and saline conditions. At the cellular level, PbCDs promoted endoreduplication and cell expansion under normal conditions, while stimulating cell division under salinity, indicating a context-dependent regulation of the cell cycle. In addition, PbCDs also improved photosynthetic performance by increasing chlorophyll content, net photosynthetic rate, stomatal conductance, and PSII efficiency, thereby alleviating salt-induced inhibition. Furthermore, PbCDs reduced ROS accumulation and lipid peroxidation while activating key antioxidant enzymes (SOD, POD, CAT, APX), maintaining cellular redox homeostasis under salt stress. Collectively, these findings revealed that PbCDs confer salt tolerance in tomatoes through dual regulation of growth and stress tolerance: enhancing photosynthetic efficiency and reinforcing antioxidant capacity while modulating cell cycle progression. This study expanded the potential applications of biomass-derived CDs and indicated that mushroom stipe base is a sustainable precursor for nanomaterial development to improve crop productivity in saline soils.

## Data Availability

The original contributions presented in this study are included in the article. Further inquiries can be directed to the corresponding authors.
